# Predictive biomarkers for the efficacy of nivolumab as ≥ 3^rd^-line therapy in patients with advanced gastric cancer: a subset analysis of ATTRACTION-2 phase III trial

**DOI:** 10.1186/s12885-022-09488-2

**Published:** 2022-04-09

**Authors:** Jwa Hoon Kim, Min-Hee Ryu, Young Soo Park, Jungeun Ma, Sun Young Lee, Deokhoon Kim, Yoon-Koo Kang

**Affiliations:** 1 Department of Oncology, Asan Medical Center, University of Ulsan College of Medicine, 88 Olympic-ro 43-gil, Songpa-gu, Seoul, 05505 Republic of Korea; 2 Division of Oncology/Hematology, Department of Internal Medicine, Korea University Anam Hospital, Korea University College of Medicine, Seoul, Republic of Korea; 3 Department of Pathology, Asan Medical Center, University of Ulsan College of Medicine, Seoul, Republic of Korea; 4 Asan Medical Center, Asan Institute for Life Sciences, University of Ulsan College of Medicine, Seoul, Republic of Korea

**Keywords:** Advanced gastric cancer, Nivolumab, Predictive biomarkers, Survival

## Abstract

**Purpose:**

The phase 3 ATTRACTION-2 study demonstrated that nivolumab monotherapy improved survival compared to placebo in patients with pretreated advanced gastric cancer (AGC). However, the efficacy of nivolumab seems to be limited to a subset of patients.

**Materials and methods:**

The predictive values of blood neutrophil–lymphocyte ratio (NLR), serum Na, PD-L1 expression, MSI status, tumor EBV infection, and tumor mutation burden (TMB) were investigated in patients with AGC refractory to ≥2 lines of chemotherapy enrolled from Asan Medical Center in ATTRACTION-2 study.

**Results:**

All 45 patients were analyzed; nivolumab (*n* = 28) and placebo (*n* = 17) groups. The objective response rate, median progression-free survival (PFS), and overall survival (OS) were 16.7%, 1.6 months, and 8.1 months in nivolumab group and 0%, 1.6 months and 6.5 months in placebo group. When comparing nivolumab with the placebo group, tumor PD-L1 expression, blood NLR, and serum Na were significant predictive factors of PFS and OS. A multivariate analysis revealed that PD-L1 ( +) and low NLR (≤ 2.9, median) were associated with better PFS. In the nivolumab group, PD-L1 ( +), low NLR, and normal Na (≥ 135 mmol/L) were associated with higher response and disease control rates, while tumor EBV infection and TMB were not.

**Conclusion:**

Tumor PD-L1 expression, blood NLR, and serum Na could be predictive biomarkers for the efficacy of nivolumab in previously treated cases of AGC.

**Supplementary Information:**

The online version contains supplementary material available at 10.1186/s12885-022-09488-2.

## Introduction

Immune checkpoint inhibitors increase antitumor T cell activity by inhibiting immune checkpoints including programmed death-1 (PD-1)/PD ligand 1 (PD-L1) [1]. The overexpression of immune checkpoints is associated with the escape mechanism of host immunity and has been observed in various types of cancer [1]. Nivolumab and pembrolizumab are human IgG4 monoclonal antibodies targeting PD-1 that are well established to improve survival in multiple solid tumors with a durable response [2–5]. In this aspect, several phase I/II trials [6–8] have reported the antitumor activity of nivolumab or pembrolizumab in advanced gastric or gastroesophageal junction cancer (G/GEJ cancer).

The ATTRACTION-2 study was the first randomized phase III trial demonstrating the efficacy of nivolumab vs. placebo in patients with advanced G/GEJ cancer refractory to ≥ 2 lines of chemotherapy (median overall survival [OS], 5.26 vs. 4.14 months; hazard ratio [HR], 0.63, *P* < 0.001 and median progression-free survival [PFS], 1.61 vs. 1.45 months; HR, 0.60, *P* < 0.001) [9]. Although the primary endpoint of OS was met, the objective response rate (ORR) was only 11.2% and no benefit in cases of rapid progression was observed in half of the study patients. These findings indicated that the efficacy of nivolumab may be limited to a certain population of patients, raising a need to identify predictive markers to select patients who can benefit from nivolumab.

Recently, the post hoc analysis from the ATTRACTION-2 study has been reported. This study explored the baseline clinical characteristics of the patients and extracted double factor combinations (serum Na and white blood cell count or neutrophil–lymphocyte ratio [NLR]) for prediction of benefit from nivolumab [10]. The triple factor combination, including age, peritoneal metastasis, and serum Na, also showed the significant association with benefit from nivolumab [10]. A high blood NLR has been reported as a poor prognostic factor in a variety of cancers treated with chemotherapy [11] and also with immune checkpoint inhibitors [12]. Hyponatremia (hypoNa, serum Na < 135 mmol/L), although relatively obscure, has been suggested as a poor prognostic factor in many cancer types [13–15]. This clinical classification is meaningful because these are easily available to clinicians in daily practice. However, it would be more valuable if these factors were analyzed together with potential biomarkers, including tumor PD-L1 expression, tumor microsatellite instability (MSI) status, tumor Epstein-Barr virus (EBV) infection, and tumor mutation burden (TMB), which were previously reported as promising biomarkers in immunological perspective. Higher ORRs with pembrolizumab were observed in PD-L1 positive (combined positive score [CPS] ≥ 1%) or MSI-high gastric cancer [7] and other various solid tumors [1, 16]. According to The Cancer Genome Atlas [1], EBV-positive tumors are associated with a high level of PD-L1 expression, suggesting that EBV infection could be a potential biomarker for immunotherapy. TMB has also been investigated as a predictor of immunotherapy response [17], and a high TMB in the tumor has been associated with a favorable response to immunotherapy [18].

To date, clear standards for selecting patients expected to exhibit optimal efficacy with nivolumb remain unelucidated. Tissues required for the molecular analyses were collected only in 40% of the patients in the ATTRACTION-2 study, thereby limiting the evaluation of tumor tissue-based biomarkers for predicting the efficacy of nivolumab. This study is a subset analysis with patients enrolled in the ATTRACTION-2 study from a single institution, Asan Medical Center (AMC). Given the advantages of available tissues, consistent management, well-controlled data quality by continuous involvement of investigators throughout the study as a single institution study and an optimal two-armed design by including a control group for evaluating the predictive values, this study aimed to investigate the predictive values of potential biomarkers such as tumor PD-L1 expression, tumor MSI status, tumor EBV infection, TMB, blood NLR, and serum Na to provide objective guidance in identifying patients with clinical benefits to nivolumab.

## Materials and methods

### Patients

The ATTRACTION-2 phase III trial was previously reported as a randomized, double-blind, placebo-controlled study at 49 clinical sites in Asia and enrolled patients with advanced G/GEJ cancer refractory to ≥ 2 lines of chemotherapy [9]. The eligibility criteria and trial design were previously described [9]. AMC participated in the ATTRACTION-2 phase III trial as one of the study sites, and the present study includes these 45 AMC patients. The study protocol was approved by the Institutional Review Board of the AMC and was conducted in accordance with the Declaration of Helsinki and good clinical practice.

### Laboratory values at baseline

Blood tests were performed prior to the patient receiving nivolumab or placebo treatment. The baseline blood NLR was calculated by dividing the absolute neutrophil count by the absolute lymphocyte count. The cut-off value for low vs. high NLR was the median value of the AMC patients (2.9). The cut-off value for hypoNa vs. normal serum Na at baseline was the lower limit of the reference value (< 135 mmol/L).

### Tumor tissue collection

Primary or metastatic tumor tissues that were obtained from endoscopic or other biopsy or surgery before the nivolumab or placebo treatment were used to analyze PD-L1 expression, MSI status, EBV infection, and TMB. Identical whole-slide sections were used for PD-L1 and MSI immunohistochemistry (IHC) staining, EBV in situ hybridization (ISH), and next-generation sequencing (NGS). Insufficient or inadequate tissues were substituted with other available tissues prior to the nivolumab or placebo treatment.

### PD-L1 immunohistochemistry

IHC staining was performed on a Dako Autostainer Link 48 system (Agilent Technologies) using a Dako PD-L1 IHC 22C3 pharmDx kit (Agilent Technologies). The level of PD-L1 protein expression was determined using the CPS, which was calculated as the number of PD-L1-stained cells (tumor cells, lymphocytes, and macrophages) divided by the total number of viable tumor cells and multiplied by 100. Tumor PD-L1 positivity was defined as CPS ≥ 1%.

### MSI immunohistochemistry and EBV in situ hybridization

MSI status was determined by IHC for mismatch repair (MMR) proteins (MLH-1, MSH-2, PMS-2, and MSH-6) in formalin-fixed paraffin-embedded (FFPE) tissues. MSI-high was defined as negative staining for at least one of the MMR proteins. EBV ISH was performed in FFPE tissues using the Bench Mark XT autostainer (Ventana Medical Systems) and ISH iVIEW Blue Detection Kit (Ventana Medical Systems), according to the manufacturer’s instructions.

### Targeted next-generation sequencing

Targeted NGS was performed using the NextSeq 550 platform (Illumina) with OncoPanel AMC version 3 (OP AMCv3; Aglient Technologies) for capturing the exons of 199 cancer-related genes, 184 hotspots, and partial introns from eight genes often rearranged in cancer. The TMB was calculated as the number of non-synonymous single nucleotide variants, insertions, and deletions in the tumor exome data.

### Statistical analysis

Categorical and quantitative data were compared using the Chi-square test or Fisher’s exact test and Mann–Whitney U-test, respectively. Survival rates were estimated using the Kaplan–Meier method with the log-rank test and predictive values were evaluated by testing the interaction between each biomarker and nivolumab treatment. Cox’s proportional hazard model was used for multivariate analysis. The multivariate analysis included factors with statistical significance (defined as *P* < 0.15) in the univariate analysis. A two-sided *P* < 0.05 was considered significant.

## Results

### Patient characteristics

**Fig. 1:**
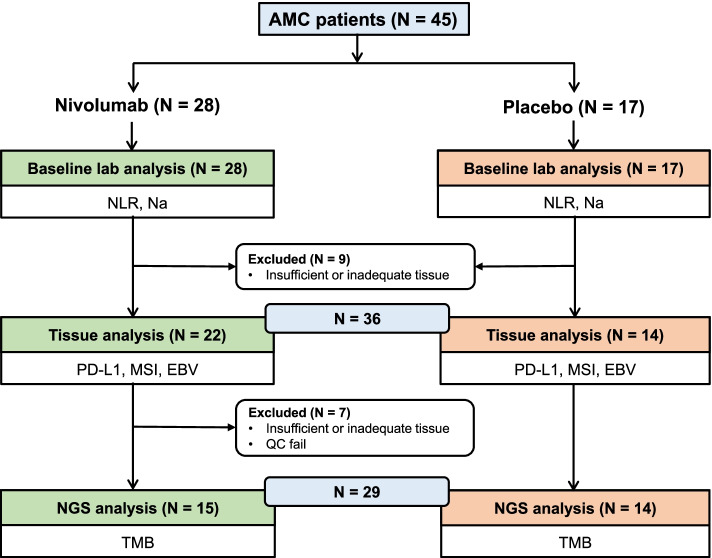
Consort flow diagram

Between November 2014 and February 2016, 493 patients were enrolled and randomly assigned to receive either nivolumab (*n* = 330) or placebo (*n* = 163) in the ATTRACTION-2 phase III trial [9]. The AMC, a single tertiary center, registered 45 patients; 28 in the nivolumab group and 17 in the placebo group, and this exploratory analysis included these 45 AMC patients. Baseline laboratory, tissue, and NGS analyses were performed in patients with available data (Fig. 1). The cut-off value of baseline blood NLR was 2.9 (median value of the AMC patients) and 135 mmol/L for baseline serum Na (reference value lower limit). Table 1 shows the baseline characteristics comparing the nivolumab and placebo groups. There were no significant differences between the nivolumab and placebo groups. Furthermore, the baseline characteristics were similar between the AMC patients and the overall patients in ATTRACTION-2 study.

**Table 1:** Baseline characteristics of the 45 AMC patients

Baseline characteristics	Total (*n* = 45), %	Nivolumab (*n* = 28), %	Placebo (*n* = 17), %
Age (median, range)	60 (23–76)	60 (23–76)	59 (33–74)
Sex (Male)	34 (75.6)	21 (75.0)	13 (76.5)
Metastatic organs			
Liver	13 (29.5)	10 (35.7)	3 (18.8)
Peritoneum	14 (31.8)	8 (28.6)	6 (37.5)
Lymph node	27 (60.0)	17 (60.7)	10 (58.8)
Others	7 (15.9)	5 (17.8)	2 (12.5)
Number of metastatic organs ≥ 2	19 (42.2)	13 (46.4)	6 (35.3)
Prior gastrectomy	26 (57.8)	14 (50.0)	12 (70.6)
HER2 positive	10 (22.2)	6 (21.4)	4 (23.5)
Treatment line			
3^rd^ line	24 (53.3)	13 (46.4)	11 (64.7)
≥ 4^th^ line	21 (46.7)	15 (56.6)	6 (35.3)
Baseline blood NLR ≤ 2.9	23 (51.1)	13 (46.4)	10 (58.8)
Baseline serum Na ≥ 135 mmol/L	38 (84.4)	24 (85.7)	14 (82.4)
**Tissue analysis**	**Total (*n* = 36), %**	**Nivolumab (*n* = 22), %**	**Placebo (*n* = 14), %**
PD-L1			
CPS < 1%	23 (63.9)	14 (63.6)	9 (64.3)
CPS ≥ 1%	13 (36.1)	8 (36.4)	5 (35.7)
CPS ≥ 10%	4 (11.2)	2 (9.1)	2 (14.3)
MSI-high	0 (0.0)	0 (0.0)	0 (0.0)
EBV-positive	6 (16.7)	5 (22.7)	1 (7.1)
**Next-generation sequencing analysis**	**Total (*n* = 29), %**	**Nivolumab (*n* = 15), %**	**Placebo (*n* = 14), %**
TMB (/Mb)	8.2 (0.0–21.3)	9.8 (0.0–21.3)	8.2 (1.6–16.4)

*AMC* Asan Medical Center, *NLR* Neutrophil–lymphocyte ratio, *MSI* Microsatellite instability, *EBV* Epstein-Barr Virus, *TMB* Tumor mutation burden

### Analysis of potential biomarkers

Baseline blood NLR and serum Na were examined in all 45 patients (Fig. 1). Low baseline blood NLR (≤ 2.9) and hypoNa at baseline serum were observed in 23 (51.1%) and 7 (15.6%) patients, respectively (Table 1). Of the 45 patients, tissue analysis was performed in 36 patients with sufficient and adequate tissues (Fig. 1), which were all obtained before the nivolumab or placebo treatment. Of the 36 tissues, 19 were acquired from endoscopic or other biopsy and 17 from surgery. The tissues were obtained from primary tumors of the stomach (*n* = 30) and metastatic lesions (*n* = 6). There were 13 patients (36.1%) with tumor PD-L1 CPS (≥ 1%) and 4 (11.2%) with tumor PD-L1 CPS (≥ 10%). Six patients (16.7%) were confirmed to have EBV ( +) gastric cancer, and MSI-high gastric cancer was not observed (Table 1). Additionally, 36 tissues passed the quality control for NGS, and 3 tissues were substituted with others due to insufficiency or inadequacy. These other 3 tissues were acquired from endoscopic biopsy of the primary stomach lesion (*n* = 1) and surgery of metastatic lesions (*n* = 2). Following exclusion due to insufficient or inadequate tissue, NGS analysis was performed on a final of 29 patients (Fig. 1). The median TMB value was 8.2 (range, 0.0–21.3) mutations/megabase (Table 1). No significant differences in baseline characteristics according to each predictive marker were observed, except for a low proportion of human epidermal growth factor receptor 2 (HER2)-positive status in patients with low baseline blood NLR.

### Predictive value of potential biomarkers in PFS and OS

With a median follow-up duration of 28.3 months in surviving patients, 43 patients (95.6%) experienced disease progression and 41 (91.1%) died from the gastric cancer. The ORR, median PFS, and OS were 16.7%, 1.6 months, and 8.1 months, respectively, in the nivolumab group and 0%, 1.6 months, and 6.5 months, respectively, in the placebo group. There were no significant differences between the AMC and overall patients in the ATTRACTION-2 study (Table S1).

**Fig. 2:**
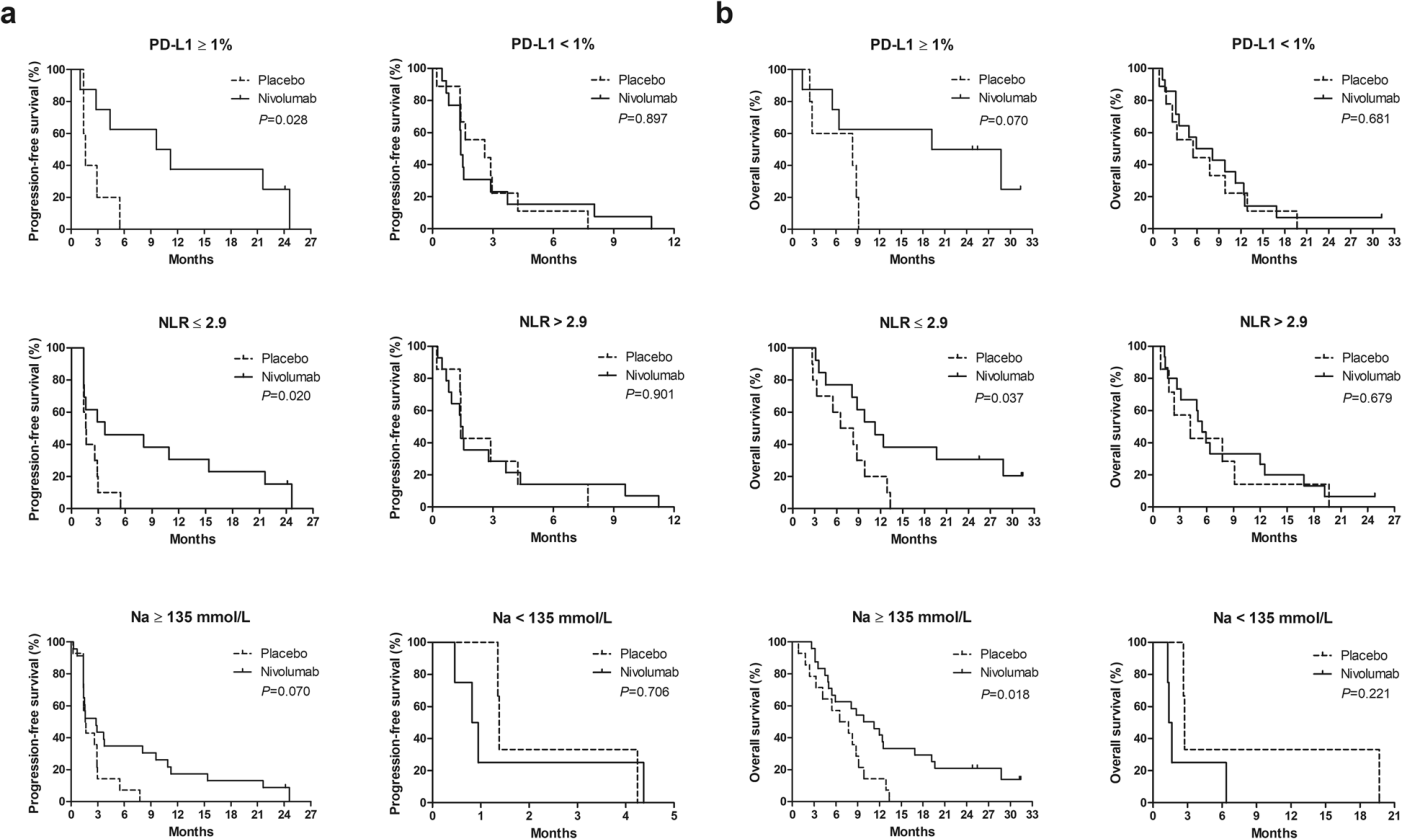
PFS (**a**) and OS (**b**) in nivolumab vs. placebo group according to each potential predictive biomarker

In patients with tumor PD-L1 CPS (≥ 1%), the median PFS was 9.6 months in the nivolumab group and 1.6 months in the placebo group (*P* = 0.028), and the median OS was 19.2 months in the nivolumab group and 8.3 months in the placebo group (*P* = 0.070). In patients with tumor PD-L1 CPS (< 1%), the median PFS was 1.4 months in the nivolumab group and 2.6 months in the placebo group (*P* = 0.897), and the median OS was 5.9 months in the nivolumab group and 5.5 months in the placebo group (*P* = 0.681) (Fig. 2) (*P*_for interaction_ = 0.003).

In patients with low baseline blood NLR, the median PFS was 3.7 months in the nivolumab group and 1.6 months in the placebo group (*P* = 0.020), and the median OS was 11.2 months in the nivolumab group and 6.5 months in the placebo group (*P* = 0.037). In patients with high baseline blood NLR (> 2.9), the median PFS was 1.4 months in the nivolumab group and 1.4 months in the placebo group (*P* = 0.901), and the median OS was 5.5 months in the nivolumab group and 4.2 months in the placebo group (*P* = 0.679) (Fig. 2) (*P*_for interaction_ = 0.007).

In patients with normal baseline serum Na (≥ 135 mmol/L), the median PFS was 2.8 months in the nivolumab group and 1.6 months in the placebo group (*P* = 0.070), and the median OS was 9.8 months in the nivolumab group and 6.5 months in the placebo group (*P* = 0.018). In patients with hypoNa at baseline serum, the median PFS was 0.8 months in the nivolumab group and 1.4 months in the placebo group (*P* = 0.706), and the median OS was 1.4 months in the nivolumab group and 2.7 months in the placebo group (*P* = 0.221) (Fig. 2) (*P*_for interaction_ = 0.026).

### Univariate and multivariate analyses for PFS and OS

Table S2 summarizes the univariate and multivariate analyses for PFS and OS. After multivariate analysis for PFS, low baseline blood NLR was associated with better PFS (HR 0.56, 95% confidence interval [CI] 0.30–1.04, *P* = 0.067). After multivariate analysis for OS, HER2 status (positive vs. negative) (HR 0.12, 95% CI 0.04–0.37, *P* < 0.001), treatment line (3^rd^ line vs. ≥ 4^th^ line) (HR 0.31, 95% CI 0.14–0.66, *P* = 0.003), baseline blood NLR (≤ 2.9 vs. > 2.9) (HR 0.34, 95% CI 0.17–0.70, *P* = 0.003), and baseline serum Na (≥ 135 vs. < 135 mmol/L) (HR 0.16, 95% CI 0.05–0.45, *P* = 0.001) remained as significant factors.

In the 36 patients where tissue was available for analysis (Table 2), baseline blood NLR (≤ 2.9 vs. > 2.9) (HR 0.46, 95% CI 0.22–0.95, *P* = 0.037) and tumor PD-L1 CPS (≥ 1% vs. < 1%) (HR 0.32, 95% CI 0.14–0.72, *P* = 0.006) remained as significant factors after multivariate analysis for PFS. For OS, age (≥ 65 years vs. < 65 years) (HR 0.33, 95% CI 0.14–0.78, *P* = 0.012) and tumor PD-L1 CPS (≥ 1% vs. < 1%) (HR 0.44, 95% CI 0.20–0.97, *P* = 0.040) remained as significant factors after multivariate analysis.

**Table 2:** Univariate and multivariate analyses for PFS and OS in the 36 patients with available tissue analysis results

	**Progression-free survival**				**Overall survival**			
	Univariate analysis		Multivariate analysis		Univariate analysis		Multivariate analysis	
	HR (95% CI)	*P*-value	HR (95% CI)	*P*-value	HR (95% CI)	*P*-value	HR (95% CI)	*P*-value
Age (≥ 65 years vs. < 65 years)	0.78 (0.36–1.70)	0.534			0.39 (0.17–0.92)	0.032	0.33 (0.14–0.78)	0.012
Sex (male vs. female)	0.76 (0.35–1.67)	0.501			0.60 (0.28–1.30)	0.197		
Tx. (nivolumab vs. placebo)	0.57 (0.27–1.22)	0.149	0.79 (0.36–1.74)	0.561	0.51 (0.25–1.05)	0.069	0.62 (0.30–1.29)	0.201
Prior gastrectomy (yes vs. no)	0.98 (0.48–1.99)	0.948			0.82 (0.40–1.69)	0.594		
HER2 (positive vs. negative)	0.77 (0.35–1.73)	0.532			0.57 (0.24–1.34)	0.198		
Number of metastatic organs (< 2 vs. ≥ 2)	0.69 (0.35–1.39)	0.303			0.85 (0.42–1.72)	0.657		
Treatment line (3^rd^ line vs. ≥ 4^th^ line)	0.69 (0.33–1.42)	0.311			0.57 (0.26–1.24)	0.158		
Baseline blood NLR (≤ 2.9 vs. > 2.9)	0.57 (0.28–1.15)	0.115	0.46 (0.22–0.95)	0.037	0.60 (0.30–1.22)	0.160		
Baseline serum Na (≥ 135 mmol/L vs. < 135 mmol/L)	0.39 (0.16–0.93)	0.033	0.50 (0.19–1.30)	0.156	0.39 (0.17–0.92)	0.031	0.82 (0.28–2.39)	0.721
Tumor PD-L1 CPS (≥ 1% vs. < 1%)	0.37 (0.17–0.83)	0.016	0.32 (0.14–0.72)	0.006	0.56 (0.26–1.20)	0.136	0.44 (0.20–0.97)	0.040
Tumor EBV (positive vs. negative)	0.61 (0.24–1.60)	0.312			0.89 (0.37–2.18)	0.803		

*HR* Hazard ratio, *CI* Confidence interval, *Tx* Treatment, *NLR* Neutrophil–lymphocyte ratio, *EBV* Epstein-Barr Virus

### Evaluation of response and PFS with nivolumab according to predictive biomarkers

Table 3 presents the ORR and DCR with nivolumab in patients with measurable disease and tissue analysis compared according to the potential biomarkers. When comparing the ORR and DCR with nivolumab according to each potential biomarker to their counterparts, a low baseline blood NLR, normal baseline serum Na, and tumor PD-L1 CPS (≥ 1%) were associated with a higher ORR and DCR, whereas tumor EBV infection and a high TMB in the tumor were not.

**Table 3:** Response to nivolumab in 24 patients with measurable disease according to the potential predictive biomarkers

	**Objective response rate (n, %)**	**Disease control rate (n, %)**
Baseline blood NLR		
> 2.9 (*n* = 14)	1 (7.1)	5 (35.7)
≤ 2.9 (*n* = 10)	3 (30)	6 (60.0)
Baseline serum Na		
< 135 mmol/L (*n* = 4)	0 (0.0)	1 (25.0)
≥ 135 mmol/L (*n* = 20)	4 (20.0)	10 (50.0)
Tumor PD-L1		
CPS < 1% (*n* = 10)	1 (10.0)	2 (20.0)
CPS ≥ 1% (*n* = 8)	3 (37.5)	7 (87.5)
CPS ≥ 10% (*n* = 2)	1 (50.0)	2 (100.0)
Tumor EBV		
Negative (*n* = 13)	3 (23.1)	6 (46.2)
Positive (*n* = 5)	1 (20.0)	3 (60.0)
Tumor mutation burden		
≤ 8.2/Mb (*n* = 6)	1 (16.7)	3 (50.0)
> 8.2/Mb (*n* = 6)	2 (33.3)	3 (50.0)

*NLR* Neutrophil–lymphocyte ratio, *EBV* Epstein-Barr Virus

**Fig. 3:**
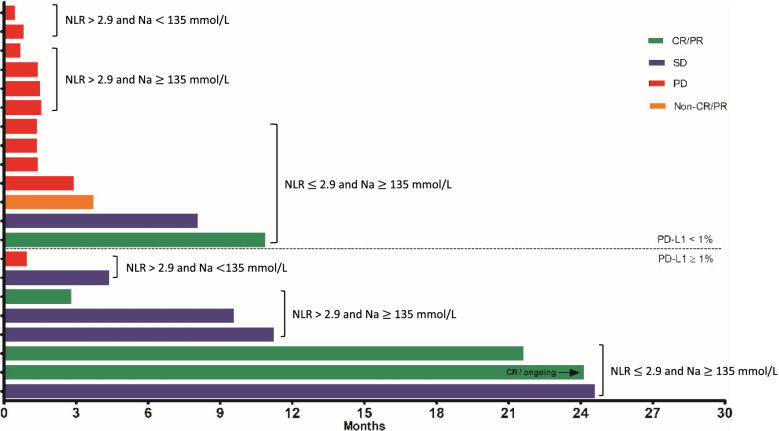
PFS of individual patients in the nivolumab group according to tumor PD-L1 expression, baseline blood NLR, and serum Na

In Fig. 3, response and PFS following nivolumab administration are shown according to baseline blood NLR, serum Na, and tumor PD-L1 in 21 patients with available tissue analysis in the nivolumab group. Seven out of the eight (87.5%) patients with tumor PD-L1 CPS (≥ 1%) showed more than stable disease with a durable PFS. Three patients with all 3 predictive biomarkers (low baseline blood NLR, normal baseline serum Na, and tumor PD-L1 CPS [≥ 1%]) exhibited approximately 2 years of PFS, with one of them showing a complete response. Two patients with tumor PD-L1 CPS (≥ 1%) but high NLR and hypoNa at baseline serum showed shorter PFS, and early progressive disease was observed in one of these patients, with a high NLR of 12 and hypoNa at baseline serum. In the 13 patients with tumor PD-L1 CPS (< 1%), 10 (76.9%) showed progressive disease. Still, two patients with low baseline blood NLR and normal baseline serum Na showed a partial response and stable disease with a durable PFS (≥ 6 months) despite tumor PD-L1 CPS (< 1%).

### Treatment-related adverse events with nivolumab

Treatment-related adverse events (TRAE) with nivolumab are shown in Table S3. The most common TRAE of any grade was pruritis (*n* = 9, 32.1%) and there were two grade 3 or 4 TRAE; fatigue (*n* = 1, 3.6%) and anorexia (*n* = 1, 3.6%). Common TRAE (> 5%) of any grade included flu-like syndrome (*n* = 5, 17.9%), AST or ALT elevation (*n* = 5, 17.9%), anorexia or nausea (*n* = 5, 17.9%), diarrhea (*n* = 4, 14.3%), skin rash (*n* = 3, 10.7%), and hyperglycemia (*n* = 3, 10.7%). One patient (3.6%) had hypothyroidism.

## Discussion

The current study evaluated the predictive values of potential biomarkers for the efficacy of nivolumab in advanced G/GEJ cancer that had been heavily treated with chemotherapy. A significant efficacy of nivolumab compared to placebo was observed in patients with tumor PD-L1 (CPS ≥ 1%), low baseline blood NLR, and normal baseline serum Na in terms of PFS and OS, while no differences in efficacy were observed under the opposite conditions (tumor PD-L1 CPS < 1%, high NLR, and hypoNa at baseline serum). After multivariate analysis, low baseline blood NLR was revealed as a significant factor for better PFS and OS, and normal baseline serum Na were also significant factors for OS. In the 36 AMC patients where tissue analysis results were available, tumor PD-L1 (CPS ≥ 1%) and low baseline blood NLR were significantly associated with better PFS in the multivariate analysis. In 24 AMC patients with measurable lesion and available tissue analysis results, PD-L1 (CPS ≥ 1%) as well as low blood NLR and normal serum Na at baseline were associated with higher ORR and DCR, while tumor EBV infection and high TMB in the tumor were not. This study is a worthy study, despite a subgroup study, to complement the limitation of the post-hoc analysis of the ATTRACTION-2 study [10], which did not include tumor-tissue based biomarkers analysis. The predictive value of both baseline blood NLR and serum Na, which were extracted as double-factor combination for prediction of benefit from nivolumab in the post-hoc analysis [10], was confirmed and could be considered to be robust, given these factors remained significant after multivariate analysis with tumor tissue-based biomarkers. Furthermore, the predictive values of tumor tissue-based biomarkers, previously explored as promising, could be validated in this study.

PD-L1 as an immune checkpoint is a reasonable biomarker for predicting the treatment response to anti-PD-1 or anti-PD-L1 therapies. In the KEYNOTE-059 phase II study, patients with PD-L1 positive (CPS ≥ 1%) gastric cancer had a higher ORR of 15.5% than patients with PD-L1 negative (CPS < 1%) gastric cancer, with an ORR of 6.4% when treated with pembrolizumab as $\ge$ 3^rd^-line therapy [7]. Similarly, in the post-hoc analysis of the KEYNOTE-061 phase III study, pembrolizumab significantly improved OS compared with paclitaxel as 2^nd^-line therapy in patients with PD-L1 positive (CPS ≥ 10%) advanced gastric cancer, whereas PFS and OS curves between pembrolizumab and paclitaxel groups crossed in patients with PD-L1 positive (CPS ≥ 1%) gastric cancer [19]. These results suggested that although pembrolizumab was not superior to paclitaxel as a 2^nd^-line therapy in the overall patients, it exhibited better survival in patients with higher tumor PD-L1 expression (CPS ≥ 10%) compared with paclitaxel [19]. In line with these findings, our results demonstrated that PD-L1 positive (CPS ≥ 1%) gastric cancer was associated with an improved survival outcome with nivolumab, confirming the predictive value of tumor PD-L1 expression.

The PD-L1 positive (CPS ≥ 1%) gastric cancer was reported in approximately 60% in the KEYNOTE-059 and KEYNOTE-061 studies using the same CPS with 22C3 pharmDx assay and 18.2% of the KEYNOTE-061 study was PD-L1 positive (CPS ≥ 10%) gastric cancer. Although these rates do not match thoroughly with that of this study, survival benefit with immunotherapy according to the intensity of tumor PD-L1 expression was consistent. Notably, however, no difference in OS was observed according to tumor PD-L1 expression status in the previous analysis of ATTRACTION-2 phase III trials in less than half of the subset of patients with available tissue (approximately 40%). The difference in methodology may contribute to this discordance. The tumor proportion score (TPS) method with a 28–8 pharmDx assay was used in the analysis of the ATTRACTION-2 phase III trial, whereas the CPS method with a 22C3 pharmDx assay was used in this study. Our study also could not observe significant predictive value of tumor PD-L1 expression using TPS (≥ 1%) with a 22C3 pharmDx assay for the response of nivolumab (Figure S1). Since the CPS method can detect the PD-L1-staining immune cells, which play a crucial role in the tumor microenvironment, this method may exhibit more robust and reproducible results to predict the response to immune checkpoint inhibitors in patients with G/GEJ cancer [20].

Along with tumor PD-L1 expression, a low baseline blood NLR was also associated with higher ORR and DCR and was noted as a significant factor for better PFS and OS in multivariate analyses. Despite many reports concerning the prognostic value of baseline blood NLR in advanced gastric cancer treated with chemotherapy [11] or immune checkpoint inhibitors [12], most of the studies were designed as single-arm studies, assessing its prognostic value within patients receiving anti-cancer treatment only. This limits the adequate evaluation of its predictive role compared with other treatments or placebo. In this study, by comparing the nivolumab group with the placebo group, the predictive role of NLR was clearly shown, as nivolumab significantly improved PFS and OS in patients with low baseline blood NLR, while no such differences were detected in patients with high baseline blood NLR. Moreover, when the PFS was examined in the nivolumab group, some distinct features were noted according to the baseline blood NLR level (Fig. 3). Despite patients with tumor PD-L1 CPS (≥ 1%) showing a relatively long PFS, one patient with a high baseline blood NLR of 12 showed a very short PFS of 0.95 months, whereas in two patients with low baseline blood NLR (2.7 and 1.2), a relatively long PFS with a durable response was observed despite tumor PD-L1 CPS (< 1%). These results may further support the value of baseline blood NLR in predicting the response with nivolumab along with other markers.

This study revealed that hypoNa significantly lowered the efficacy of nivolumab. The incidence of hypoNa in in gastrointestinal cancers is approximately 10% and is associated with poor survival in various cancers [13, 15]. However, the cause of hypoNa and its association with survival outcomes are unclear. HypoNa may simply be regarded to represent poor general condition and this may explain, at least in part, its association with unfavorable clinical outcomes. However, a more specific role of Na in the immune system has also been investigated. A high concentration of Na can enhance inflammatory M1-associated macrophages and T helper-17 cells and reduce regulatory T cells and M2 macrophages [21–23]. This impact of Na on the function of immune cells can subsequently influence the onset and progression of many disease including cancers [21–23]. Although still elusive, we suggest that low baseline serum Na levels can contribute to a poor response to nivolumab.

Tumor EBV infection is characterized by tumor PD-L1 expression and intra- or peritumoral immune cell infiltration. EBV ( +) tumors can be considered as candidates for immunotherapy. To date, however, clinical data regarding the response of EBV ( +) tumors to immunotherapy is lacking. One study in advanced gastric cancer treated with pembrolizumab as ≥ 2^nd^-line therapy has provided clinical evidence showing that all EBV ( +) gastric cancer (6 of 61, 9.8%) exhibited ORR of 100% and tumor PD-L1 CPS (≥ 1%) [24]. However, the sample size may be too small to conclude the association of EBV positivity with the response to immunotherapy and tumor PD-L1 expression. In this study, although our six patients with EBV ( +) gastric cancer (5 in the nivolumab and 1 in the placebo group) also showed a trend toward a high percentage (4/6, 66.7%) of tumor PD-L1 CPS (≥ 1%) compared with those of patients with EBV ( −) gastric cancer (9/30, 30%), 2 out of 5 EBV ( +) patients in the nivolumab group showed early progressive disease (Figure S2). The interpretation requires caution due to the small sample size and different types of immune checkpoint inhibitors.

The data regarding TMB and tumor MSI status was limited for the proper evaluation of the results from this study. There was no MSI-high gastric cancer, and TMB could only be calculated in a small number of patients due to insufficient or inadequate tissues. Although the median TMB value was used to determine low vs. high TMB in this study, the cut-off value varied between studies without consensus and could result in different interpretations [25, 26]. The predictive value of TMB has been well established in melanoma [27] and lung cancer [28, 29], which always represent the highest TMB across the various cancers [25, 26]. The strong correlation of TMB with the efficacy of immunotherapy can be considered in tumor types with considerable TMB [25]. Additionally, different methods between comprehensive genomic profiling and whole exome sequencing for measuring TMB need to be considered. Although comprehensive genomic profiling using the FoundationOne assay (Cambridge) agrees with WES, variations increase as the number of megabases sequenced decreases, especially at lower levels of TMB [25]. Also, the association between comprehensive genomic profiling and whole exome sequencing remains unclear in advanced gastric cancer, and further evidence regarding the predictive role of TMB in advanced gastric cancer should be accumulated.

A prospective study reported several characteristics of responders to nivolumab in patients with advanced gastric cancer by using single-arm design [30]. High ORR and favorable PFS were associated with good performance, MSI-high, and tumor PD-L1 expression status, whereas EBV ( +) and TMB were not [30]. Likewise, this study highlights the importance of PD-L1 again and similar findings on EBV ( +) or TMB was observed in both studies [30], implicating the need for further evaluation of EBV and TMB in advanced gastric cancer.

The strength of our study is that this AMC data stems from the ATTRACTION-2 trial, which was the first randomized phase III trial with positive results and this unique dataset is not reproducible because it is ethically unlikely that there will be a prospective controlled study in the future comparing nivolumab alone with placebo since the ATTRACION-2 trial. Despite small sample size, this study adequately analyzed predictive values of multiple biomarkers using two-armed design. Further, larger studies to confirm the value of biomarkers are required to provide solid evidence.

The efficacy of nivolumab has been similarly shown in 45 AMC patients included in the ATTRACTION-2 study as in the overall ATTRACTION-2 study patients. Despite the small sample size, these results suggest that tumor PD-L1 expression, baseline blood NLR, and serum Na could be potential predictive biomarkers for the efficacy of nivolumab in previously treated advanced gastric cancer.

## Supplementary Information

**Additional file 1:** **Table S1.** Clinical outcomes of AMC and overall patients in ATTRACTION-2 study. **Table S2.** Univariate and multivariate analyses for PFS and OS in the 45 AMC patients. **Table S3.** Treatment-related adverse events with nivolumab. **Figure S1.** PFS (a) and OS (b) in nivolumab vs. placebo group according to PD-L1 TPS. **Figure S2.** PFS of five patients with EBV-positive-advanced gastric cancer.

## Data Availability

The datasets generated during and/or analyzed during the current study are available from the corresponding author (YKK) on reasonable request. The data are not publicly available due to them containing information that could compromise research participant privacy/consent.

## Abbreviations

AGC: Advanced gastric cancer
NLR: Neutrophil–lymphocyte ratio
TMB: Tumor mutation burden
PFS: Progression-free survival
OS: Overall survival
PD-1: Programmed death-1
PD-L1: PD ligand 1
G/GEJ cancer: Gastric or gastroesophageal junction cancer
HR: Hazard ratio
ORR: Objective response rate
DCR: Disease control rate
hypoNa: Hyponatremia
MSI: Microsatellite instability
EBV: Tumor Epstein-Barr virus
CPS: Combined positive score
AMC: Asan Medical Center
IHC: Immunohistochemistry
ISH: In situ Hybridization
NGS: Next-generation sequencing
MMR: Mismatch repair
FFPE: Formalin-fixed paraffin-embedded
HER2: Human epidermal growth factor receptor 2
CI: Confidence interval
TPS: Tumor proportion score

## References

[1] Cancer Genome Atlas Research Network (2014). Comprehensive molecular characterization of gastric adenocarcinoma. Nature.

[2] Ferris RL, Blumenschein G, Fayette J, Guigay J, Colevas AD, Licitra L (2016). Nivolumab for recurrent squamous-cell carcinoma of the head and neck. N Engl J Med.

[3] Motzer RJ, Escudier B, McDermott DF, George S, Hammers HJ, Srinivas S (2015). Nivolumab versus everolimus in advanced renal-cell carcinoma. N Engl J Med.

[4] Herbst RS, Baas P, Kim DW, Felip E, Pérez-Gracia JL, Han JY (2016). Pembrolizumab versus docetaxel for previously treated, PD-L1-positive, advanced non-small-cell lung cancer (KEYNOTE-010): a randomised controlled trial. Lancet.

[5] Bellmunt J, de Wit R, Vaughn DJ, Fradet Y, Lee JL, Fong L (2017). Pembrolizumab as second-line therapy for advanced urothelial carcinoma. N Engl J Med.

[6] Muro K, Chung HC, Shankaran V, Geva R, Catenacci D, Gupta S (2016). Pembrolizumab for patients with PD-L1-positive advanced gastric cancer (KEYNOTE-012): a multicentre, open-label, phase 1b trial. Lancet Oncol.

[7] Fuchs CS, Doi T, Jang RW, Muro K, Satoh T, Machado M (2018). Safety and efficacy of pembrolizumab monotherapy in patients with previously treated advanced gastric and gastroesophageal junction cancer: phase 2 clinical KEYNOTE-059 trial. JAMA Oncol.

[8] Janjigian YY, Bendell J, Calvo E, Kim JW, Ascierto PA, Sharma P (2018). CheckMate-032 study: efficacy and safety of nivolumab and nivolumab plus ipilimumab in patients with metastatic esophagogastric cancer. J Clin Oncol.

[9] Kang YK, Boku N, Satoh T, Ryu MH, Chao Y, Kato K (2017). Nivolumab in patients with advanced gastric or gastro-oesophageal junction cancer refractory to, or intolerant of, at least two previous chemotherapy regimens (ONO-4538-12, ATTRACTION-2): a randomised, double-blind, placebo-controlled, phase 3 trial. Lancet.

[10] Kang YK, Morita S, Satoh T, Ryu MH, Chao Y, Kato K (2022). Exploration of predictors of benefit from nivolumab monotherapy for patients with pretreated advanced gastric and gastroesophageal junction cancer: post hoc subanalysis from the ATTRACTION-2 study. Gastric Cancer.

[11] Templeton AJ, McNamara MG, Šeruga B, Vera-Badillo FE, Aneja P, Ocana A (2014). Prognostic role of neutrophil-to-lymphocyte ratio in solid tumors: a systematic review and meta-analysis. J Natl Cancer Inst.

[12] Sacdalan DB, Lucero JA, Sacdalan DL (2018). Prognostic utility of baseline neutrophil-to-lymphocyte ratio in patients receiving immune checkpoint inhibitors: a review and meta-analysis. Onco Targets Ther.

[13] Schutz FA, Xie W, Donskov F, Sircar M, McDermott DF, Rini BI (2014). The impact of low serum sodium on treatment outcome of targeted therapy in metastatic renal cell carcinoma: results from the international metastatic renal cell cancer database consortium. Eur Urol.

[14] Hansen O, Sørensen P, Hansen KH (2010). The occurrence of hyponatremia in SCLC and the influence on prognosis: a retrospective study of 453 patients treated in a single institution in a 10-year period. Lung Cancer.

[15] Castillo JJ, Glezerman IG, Boklage SH, Chiodo J, Tidwell BA, Lamerato LE (2016). The occurrence of hyponatremia and its importance as a prognostic factor in a cross-section of cancer patients. BMC Cancer.

[16] Le DT, Uram JN, Wang H, Bartlett BR, Kemberling H, Eyring AD (2015). PD-1 blockade in tumors with mismatch-repair deficiency. N Engl J Med.

[17] Johnson DB, Frampton GM, Rioth MJ, Yusko E, Xu Y, Guo X (2016). Targeted next generation sequencing identifies markers of response to PD-1 blockade. Cancer Immunol Res.

[18] Chan TA, Yarchoan M, Jaffee E, Swanton C, Quezada SA, Stenzinger A (2019). Development of tumor mutation burden as an immunotherapy biomarker: utility for the oncology clinic. Ann Oncol.

[19] Shitara K, Özgüroğlu M, Bang YJ, Di Bartolomeo M, Mandalà M, Ryu MH (2018). Pembrolizumab versus paclitaxel for previously treated, advanced gastric or gastro-oesophageal junction cancer (KEYNOTE-061): a randomised, open-label, controlled, phase 3 trial. Lancet.

[20] Kulangara K, Zhang N, Corigliano E, Guerrero L, Waldroup S, Jaiswal D (2019). Clinical utility of the combined positive score for programmed death ligand-1 expression and the approval of pembrolizumab for treatment of gastric cancer. Arch Pathol Lab Med.

[21] Willebrand R, Kleinewietfeld M (2018). The role of salt for immune cell function and disease. Immunology.

[22] Kleinewietfeld M, Manzel A, Titze J, Kvakan H, Yosef N, Linker RA (2013). Sodium chloride drives autoimmune disease by the induction of pathogenic T_H_17 cells. Nature.

[23] Hernandez AL, Kitz A, Wu C, Lowther DE, Rodriguez DM, Vudattu N (2015). Sodium chloride inhibits the suppressive function of FOXP3^+^ regulatory T cells. J Clin Invest.

[24] Kim ST, Cristescu R, Bass AJ, Kim KM, Odegaard JI, Kim K (2018). Comprehensive molecular characterization of clinical responses to PD-1 inhibition in metastatic gastric cancer. Nat Med.

[25] Chalmers ZR, Connelly CF, Fabrizio D, Gay L, Ali SM, Ennis R (2017). Analysis of 100,000 human cancer genomes reveals the landscape of tumor mutational burden. Genome Med.

[26] Alexandrov LB, Nik-Zainal S, Wedge DC, Aparicio SA, Behjati S, Biankin AV (2013). Signatures of mutational processes in human cancer. Nature.

[27] Van Allen EM, Miao D, Schilling B, Shukla SA, Blank C, Zimmer L (2015). Genomic correlates of response to CTLA-4 blockade in metastatic melanoma. Science.

[28] Rizvi NA, Hellmann MD, Snyder A, Kvistborg P, Makarov V, Havel JJ, Cancer immunology (2015). Mutational landscape determines sensitivity to PD-1 blockade in non-small cell lung cancer. Science.

[29] Spigel DR, Schrock AB, Fabrizio D, Frampton GM, Sun J, He J (2016). Total mutation burden (TMB) in lung cancer (LC) and relationship with response to PD-1/PD-L1 targeted therapies. J Clin Oncol.

[30] Mishima S, Kawazoe A, Nakamura Y, Sasaki A, Kotani D, Kuboki Y (2019). Clinicopathological and molecular features of responders to nivolumab for patients with advanced gastric cancer. J Immunother Cancer.

