# Supplementary material for: Predictive biomarkers for the efficacy of nivolumab as ≥ 3rd-line therapy in patients with advanced gastric cancer: a subset analysis of ATTRACTION-2 phase III trial
Source: BMC Cancer. 2022 Apr 9;22:378. doi: 10.1186/s12885-022-09488-2 (PMC8994342; doi:10.1186/s12885-022-09488-2)
Supplement: Supplementary file 1 — Additional file 1: Table S1. Clinical outcomes of AMC and overall patients in ATTRACTION-2 study. Table S2. Univariate and multivariate analyses for PFS and OS in the 45 AMC patients. Table S3. Treatment-related adverse events with nivolumab. Figure S1. PFS (a) and OS (b) in nivolumab vs. placebo group according to PD-L1 TPS. Figure S2. PFS of five patients with EBV-positive-advanced gastric cancer. [file 12885_2022_9488_MOESM1_ESM.docx]

**Table S1. Clinical outcomes of AMC and overall patients in ATTRACTION-2 study**

|  | **AMC patients** | | **Overall patients** | |
| --- | --- | --- | --- | --- |
|  | Nivolumab (n = 28) | Placebo (n = 17) | Nivolumab (n = 330) | Placebo (n = 163) |
| Median PFS | 1.6 months | 1.6 months | 1.6 months | 1.5 months |
| Median OS | 8.1 months | 6.5 months | 5.3 months | 4.1 months |
| **Measurable disease** | Nivolumab (n = 24) | Placebo (n = 13) | Nivolumab (n = 268) | Placebo (n = 131) |
| ORR | 16.7% | 0% | 11.2% | 0% |
| DCR | 45.8% | 46.2% | 40.3% | 25% |

AMC, Asan Medical Center; PFS, progression-free survival; OS, overall survival; ORR, objective response rate; DCR, disease control rate

**Table S2. Univariate and multivariate analyses for PFS and OS in the 45 AMC patients**

|  | **Progression-free survival** | | | | **Overall survival** | | | |
| --- | --- | --- | --- | --- | --- | --- | --- | --- |
|  | Univariate analysis | | Multivariate analysis | | Univariate analysis | | Multivariate analysis | |
|  | HR (95% CI) | *p*-value | HR (95% CI) | *P*-value | HR (95% CI) | *P*-value | HR (95% CI) | *P*-value |
| Age (≥65 years vs. <65 years) | 0.69 (0.35–1.36) | 0.285 |  |  | 0.45 (0.22–0.93) | 0.032 | 0.72 (0.33–1.56) | 0.402 |
| Sex (male vs. female) | 0.83 (0.41–1.70) | 0.619 |  |  | 0.61 (0.30–1.23) | 0.167 |  |  |
| Tx. (nivolumab vs. placebo) | 0.60 (0.31–1.16) | 0.126 | 0.61 (0.32–1.19) | 0.146 | 0.57 (0.30–1.08) | 0.087 | 0.63 (0.31–1.27) | 0.198 |
| Prior gastrectomy (yes vs. no) | 0.98 (0.53–1.82) | 0.952 |  |  | 0.85 (0.45–1.59) | 0.608 |  |  |
| HER2 (positive vs. negative) | 0.62 (0.39–1.75) | 0.624 |  |  | 0.55 (0.25–1.21) | 0.136 | 0.12 (0.04–0.37) | <0.001 |
| Number of metastatic organs (<2 vs. ≥2) | 1.49 (0.80–2.78) | 0.207 |  |  | 1.20 (0.65–2.25) | 0.560 |  |  |
| Treatment line (3^rd^ line vs. ≥4^th^ line) | 0.71 (0.38–1.33) | 0.287 |  |  | 0.59 (0.31–1.14) | 0.119 | 0.31 (0.14–0.66) | 0.003 |
| Baseline blood NLR (≤2.9 vs. 2.9) | 0.55 (0.29–1.04) | 0.067 | 0.56 (0.30–1.04) | 0.067 | 0.59 (0.32–1.11) | 0.101 | 0.34 (0.17–0.70) | 0.003 |
| Baseline serum Na (≥135 mmol/L vs. <135 mmol/L) | 0.46 (0.20–1.06) | 0.068 | 0.61 (0.25–1.48) | 0.277 | 0.42 (0.18–0.96) | 0.039 | 0.16 (0.05–0.45) | 0.001 |

HR, hazard ratio; CI, confidence interval; Tx, treatment; NLR, neutrophil-lymphocyte ratio

**Table S3. Treatment-related adverse events with nivolumab**

|  | Any grade | Grade 3 or 4 |
| --- | --- | --- |
| AST elevation | 5 (17.9) | 0 (0) |
| ALT elevation | 4 (14.3) | 0 (0) |
| Hyperglycemia | 3 (10.7) | 0 (0) |
| Hypothyroidism | 1 (3.6) | 0 (0) |
| Fatigue | 5 (17.9) | 1 (3.6) |
| Flu-like syndrome | 5 (17.9) | 0 (0) |
| Myalgia | 1 (3.6) | 0 (0) |
| Anorexia | 5 (17.9) | 1 (3.6) |
| Nausea | 5 (17.9) | 0 (0) |
| Diarrhea | 4 (14.3) | 0 (0) |
| Pruritis | 9 (32.1) | 0 (0) |
| Rash | 3 (10.7) | 0 (0) |

**Supplementary Figure legends:**

**Figure S1**. PFS (a) and OS (b) in nivolumab vs. placebo group according to PD-L1 TPS.


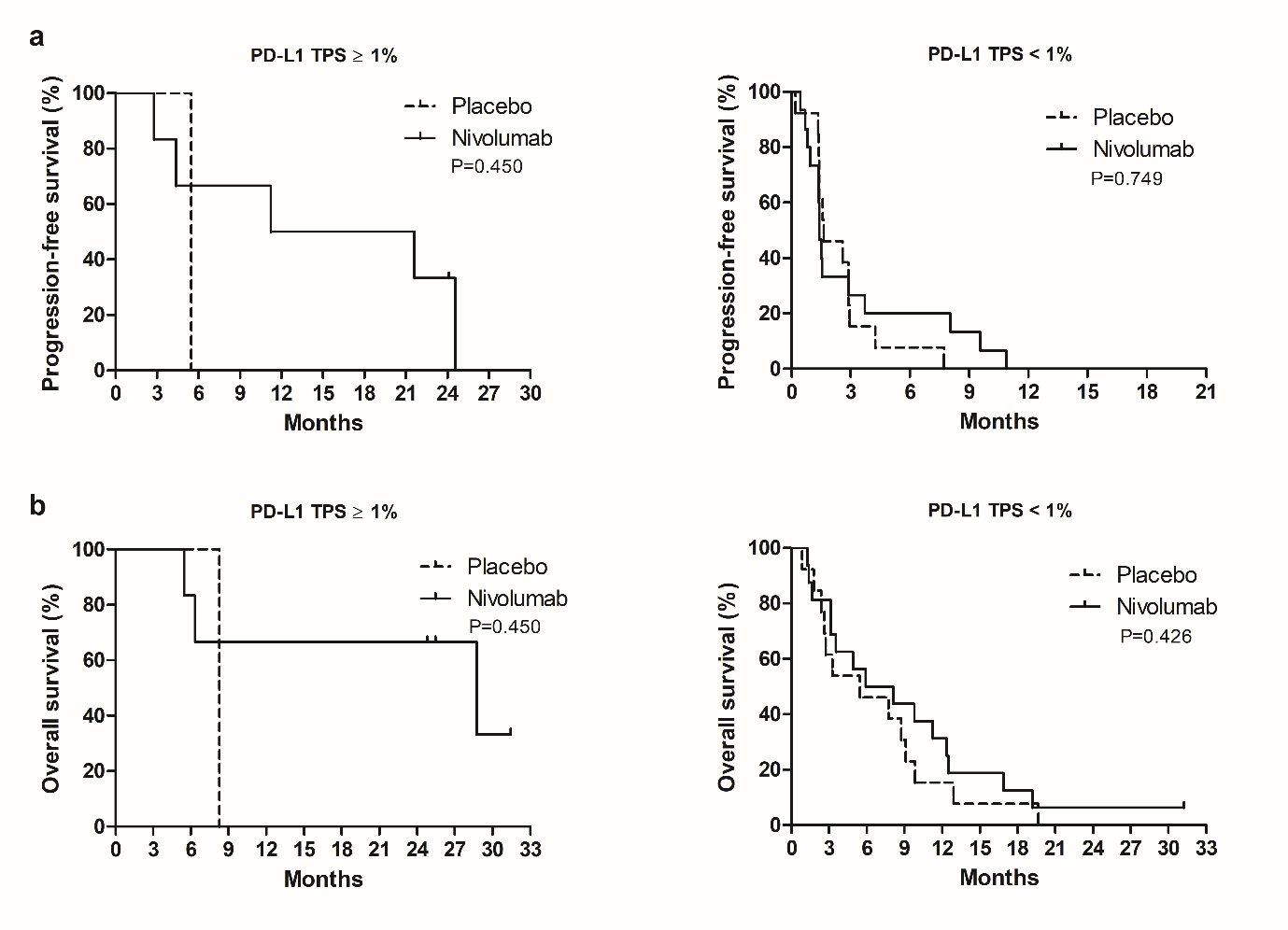


PFS, progression-free survival; OS, overall survival; TPS, tumor proportion score

**Figure S2**. PFS of five patients with EBV-positive-advanced gastric cancer.


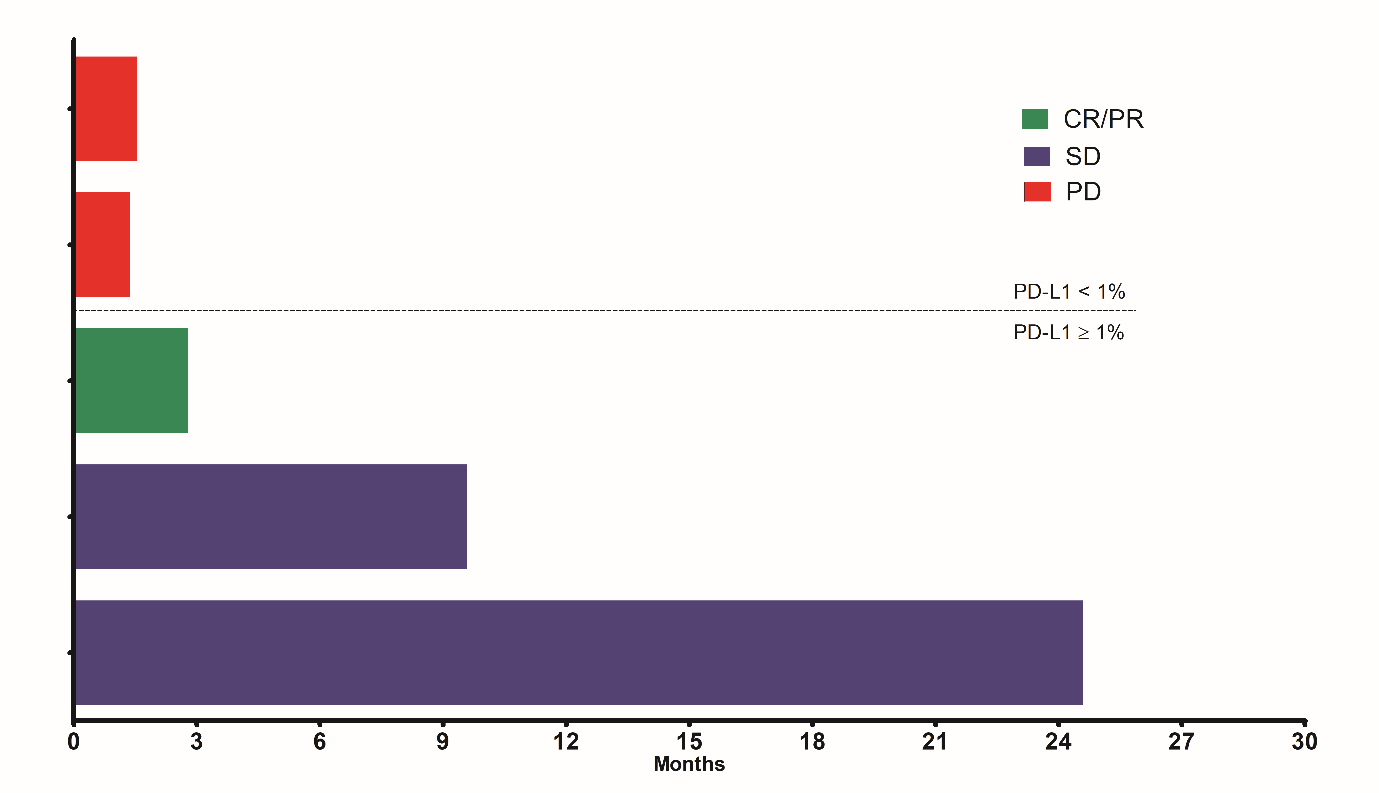


CR, complete response; PR, partial response; SD, stable disease; PD, progressive disease
